# Analysis of a Novel T1-like Phage KanT1 Reveals a Standalone SH3 Domain as a Widespread Component of *Drexlerviridae* Cell Lysis Module

**DOI:** 10.3390/ijms27093756

**Published:** 2026-04-23

**Authors:** Arina Eremina, Polina Iarema, Oksana Kotovskaya, Aleksandr Shenfeld, Alina Demkina, Kristina Ivanova, Alena Drobiazko, Daria Morozova, Konstantin Severinov, Artem Isaev

**Affiliations:** 1 The Center for Bio- and Medical Technologies, 121205 Moscow, Russia; arina17072005@gmail.com (A.E.); iaremapolina@gmail.com (P.I.); shenaleksandr@gmail.com (A.S.); alinademkina98@gmail.com (A.D.); nokris752@gmail.com (K.I.); alena.drobiazko.brex@gmail.com (A.D.); darya.d.morozova@gmail.com (D.M.); 2 Shemyakin-Ovchinnikov Institute of Bioorganic Chemistry, 117997 Moscow, Russia; 3 Institute of Gene Biology, Russian Academy of Sciences, 119334 Moscow, Russia; severik@waksman.rutgers.edu

**Keywords:** *Tunavirus*, endolysin, superinfection exclusion, comparative genomics, structure-based annotation

## Abstract

Bacteriophages are ubiquitous biological entities that profoundly influence microbiology research and biotechnology. Among coliphages, T1-like viruses (family *Drexlerviridae*) are notoriously known for their environmental stability and propensity to contaminate laboratory cultures and equipment. Despite this, the genomic features that may underlie their persistence and recurrent detection as laboratory contaminants remain insufficiently characterized. Here, we describe a novel T1-like bacteriophage, KanT1, identified as a recurrent contaminant emerging from environmental samples. Comparative genomics and phylogenetic analyses position KanT1 within the *Tunavirus* lineage, confirming its close relationship to canonical T1-like phages. Structure-informed annotation enabled the functional characterization of previously unannotated proteins, highlighting the importance of integrating structural predictions into phage genome analysis. Notably, we provide novel details regarding the distribution of superinfection exclusion cassette *cor* and identify an SH3 domain-containing protein associated with the lysis cassette. We show that SH3 is widespread, though non-universal, across *Drexlerviridae* genomes. Given the established role of SH3 domains as determinants of cell-wall binding specificity for endolysins of phages infecting Gram-positive bacteria, we propose that this protein represents an auxiliary component of the T1-like lysis module. Together, these findings expand the current understanding of T1-like phage genome organization and provide new insights into molecular features that may contribute to their broad host range and persistence in laboratory environments.

## 1. Introduction

Bacteriophages are the most abundant biological entities on Earth and play central roles in microbial ecology, evolution, and biotechnology. At the same time, phages represent a persistent and often underestimated source of contamination in microbiological laboratories, where they can uncontrollably spread, cause rapid culture collapse, and compromise reproducibility [1].

Among *Escherichia coli* phages, T1 and T1-like viruses are particularly notorious in this context. Historical and modern observations consistently indicate that these phages are unusually stable, difficult to eliminate, and capable of persisting in laboratory environments [2]. Their ability to repeatedly contaminate *E. coli* cultures has made them a long-standing practical concern, especially in workflows involving high-density bacterial growth or protein production [3]. In some instances, resolution of T1 contamination at protein-producing facilities even requires construction of genetically engineered resistant strains [4].

From a genomic perspective, T1-like phages belong to the family *Drexlerviridae*, and morphologically belong to the siphoviruses—phages with long, flexible, non-contractile tails—while some of T1 structural components, like the head and head-to-tail connector, are close to myophages [5]. *Drexlerviridae* share a conserved genome architecture combined with modular variability in host recognition and accessory functions, while the functions of ~50% of proteins, predominantly small ORFs, in the classical T1 genome remain undetermined [6,7]. It is supposed that T1-like phages use lateral tail fibers for the reversible attachment to the O-antigen as their primary receptor, while irreversible attachment and genome injection are triggered by tail tip interaction with the outer membrane protein receptors, like FhuA, LptD, BtuB, TolC, YncD, or FepA [8,9,10,11,12]. T1 also encodes a superinfection exclusion lipoprotein Cor, which blocks accessibility of the FhuA receptor early after infection starts, contributing to competitive exclusion of other phages and supporting the correct temporal pattern of T1 gene expression [12,13].

Phage T1 cell lysis module resembles a classical architecture and is composed of the pinholin, which punctures the inner membrane; SAR endolysin decomposing the peptidoglycan cell wall; and unimolecular u-spanin, which anchors into both inner and outer membranes and creates a channel promoting phage progeny release [14,15]. Endolysins require a catalytic domain to carry out their function and may additionally harbor diverse cell-wall-binding (CWB) domains, among which SH3 domains are particularly widespread. Such a modular architecture is characteristic primarily of endolysins from phages infecting Gram-positive bacteria [16]. Although examples of modular organization have also been described in endolysins of phages targeting Gram-negative hosts, the presence of SH3-like CWB domains in these systems has not yet been reported [17,18,19,20].

Despite their long-standing relevance, the molecular determinants that contribute to the robustness and persistence of T1-like phages remain incompletely understood. In this study, we characterize a novel T1-like phage, KanT1, identified as a recurrent laboratory contaminant emerging from environmental samples. Similar to the classical coliphage T1, KanT1 exploits the FhuA receptor, yet demonstrates an extended host range. Using comparative genomics, phylogenetics, and structure-guided annotation, we place KanT1 within the *Tunavirus* genus and describe its genome organization. Furthermore, we identify an SH3 domain-containing protein associated with the lysis cassette and demonstrate its widespread occurrence across *Drexlerviridae*. These findings reveal an additional layer of complexity in T1-like lysis modules.

## 2. Results and Discussion

### 2.1. Isolation, Life Cycle, and Morphology of KanT1

**Figure 1:**
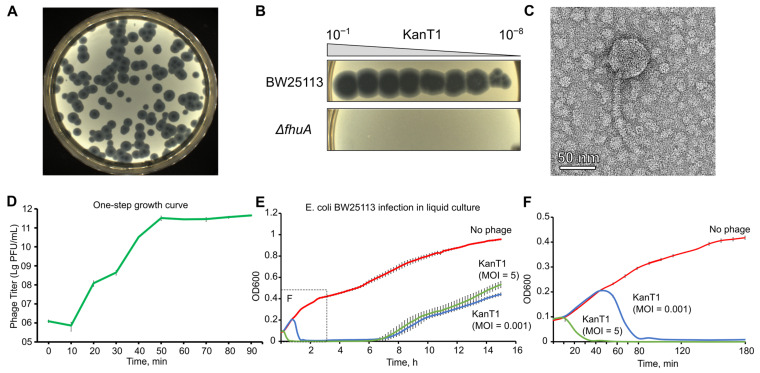
(**A**) Morphology of KanT1 plaques on an *E. coli* BW25113 bacterial lawn. (**B**) Efficiency of plaquing (EOP) of KanT1 on wild-type (wt) *E. coli* BW25113 and its *fhuA* deletion mutant. (**C**) Representative TEM image of KanT1 virion. (**D**) One-step growth curve of KanT1 in liquid LB medium at 37 °C. (**E**) Growth curves of *E. coli* BW25113 liquid cultures infected with KanT1 at low (MOI = 0.001) or high (MOI = 5) multiplicity of infection. (**F**) Zoomed-in timescale (first 3 h) showing the early growth phase in detail.

A novel phage was isolated from a river water sample, using *E. coli* BW25113 as a host. While initial screens of the water samples demonstrated a diversity of plaque morphologies, further propagation of each isolated phage resulted in the production of uniform large clear plaques with a turbid halo (Figure 1A,B), indicating the possible contamination of phage stocks with one of the environmental isolates. To better understand the nature of the contaminating phage, we isolated and sequenced its genome, identifying it as a relative of the notoriously known phage T1. The phage was obtained from a sample collected in the Parkoviy Stream in Kaliningrad, Russia (previously known as Königsberg), the birthplace of the German philosopher Immanuel Kant. To commemorate this, we named the novel phage KanT1. According to a formal nomenclature, the phage could be named as vB_EcoS_KanT1.

Phage T1 uses FhuA as a protein receptor, and we confirmed that KanT1 plaquing also depends on the FhuA presence (Figure 1B). On the lawn of its host, the phage formed plaques with a characteristic T1-like morphology: the plaques measured approximately 4.7 mm (a mean of 4.7 ± 0.6 mm) in diameter, featuring a clear, sharply defined center comprising about one-third of the total area, surrounded by a distinctly turbid halo (Figure 1A) [21]. A one-step growth curve analysis and bacterial growth dynamics of the host strain BW25113 at 37 °C revealed a highly aggressive lytic cycle with a latent period of approximately 15–20 min and a burst size of ~100 progeny particles per infected cell (Figure 1D–F) [22]. Notably, the bacterial growth was observed in the liquid culture approximately 7 h post-infection, suggesting the accumulation of receptor mutants.

Transmission electron microscopy (TEM) imaging confirmed siphovirus morphology. KanT1 possesses an icosahedral head with a diameter of 60 nm (a mean of 60 ± 4 nm) and a long, flexible, non-contractile tail measuring 155 nm (a mean of 155 ± 7 nm) in length and 9 nm (a mean of 9 ± 1 nm) in diameter (Figure 1C) [21].

### 2.2. Host Range Profiling of KanT1 Within the ECOR Collection

**Figure 2:**
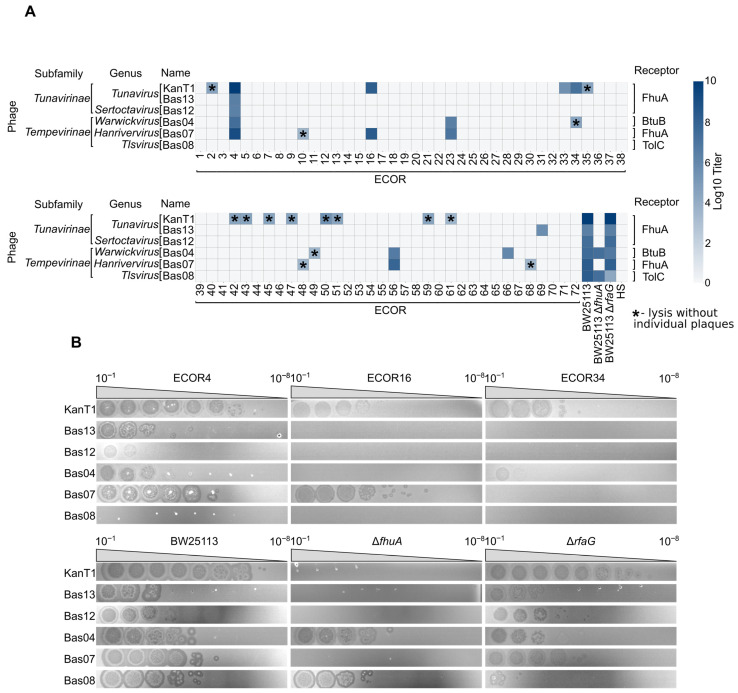
(**A**) Heatmap showing log_10_ titers of phages on a panel of *E. coli* strains, including the ECOR reference collection (ECOR1–ECOR72) producing O-antigens, laboratory strains (BW25113 and derivatives) and HS. Rows correspond to phages from different genera within the *Tunavirinae* subfamily, and columns represent bacterial hosts. Color intensity reflects phage titer, with darker shades indicating higher efficiency of plating and light/empty cells indicating resistance or absence of detectable infection. Asterisk represents lysis of the culture without visible individual phage plaques. (**B**) An efficiency of plaquing assay demonstrating phage morphology and inhibition level.

We next assessed the host range of KanT1 using a panel of *E. coli* strains from the ECOR reference collection representing the natural diversity of *E. coli* O-antigens [23], as well as a common human gut commensal strain HS [24]. We compared KanT1 plaquing efficiency with a representative set of phages from the BASEL collection, belonging to different *Drexlerviridae* genera [8]. The efficiency of plating was visualized as a log_10_ titer on a heatmap (Figure 2A,B).

KanT1 and most other T1-like phages efficiently infected the ECOR4 strain, while ECOR16 and ECOR34 supported formation of clearly visible KanT1 plaques, albeit with a reduced titer (Figure 2B). This phenotype could indicate interference from non-abortive immunity systems of the host or decreased receptor availability. Eleven other ECOR strains demonstrated the signs of lysis without clear plaque formation or produced poorly visible individual plaques. In total, 14 out of 72 strains from the collection (~20%) demonstrated some degree of sensitivity to KanT1, confirming its broad host range and an ability to infect *E. coli* strains bearing an O-antigen.

The role of later tail fibers (LTFs) of the phage T1 has not been described in detail, although in other phages LTFs mediate the first, reversible stage of adsorption to the O-antigen or lipopolysaccharide (LPS). Interestingly, an LPS was shown to be required for the infection by T1-like phage Rtp [12]. Plaquing efficiency of some *Drexlervidiae* phages from the BASEL phage collection was also shown to be reduced on BW25113 *E. coli* host with truncated LPS [8]. To investigate the requirement of the LPS for KanT1 infection, we utilized a Δ*rfaG* (Δ*waaG*) strain from the KEIO collection [25]. KanT1 titer was ~30% reduced on the lawn of this strain, and the plaque halo morphology differed, suggesting that interaction with LPS could contribute to the efficient phage adsorption. Confirming previous observation, the titer of the Bas08 was significantly reduced on truncated LPS strain Δ*rfaG* (Figure 2B). Our data demonstrate that this phage was unable to infect any of the strains from the ECOR collection, suggesting that it requires direct access to LPS for adsorption. Finally, we demonstrate that a gut commensal strain HS was resistant to all tested T1-like phages. Since *E. coli* HS supports efficient transformation and protein expression, this suggests that HS can be used as one of the alternative model strains for transformation, cloning, and protein production in T1-contaminated laboratory environments.

Overall, these results position KanT1 as a phage with a relatively broad host range, as compared to the sensitivity of the ECOR collection to other phages [26], capable of infecting multiple *E. coli* strains but restricted by protein receptor specificity and host-dependent barriers. Such a profile is characteristic of T1-like phages and supports the idea that variation in tail fiber proteins underlies host range diversification within this group.

### 2.3. Genomic Organization of the KanT1

**Figure 3:**
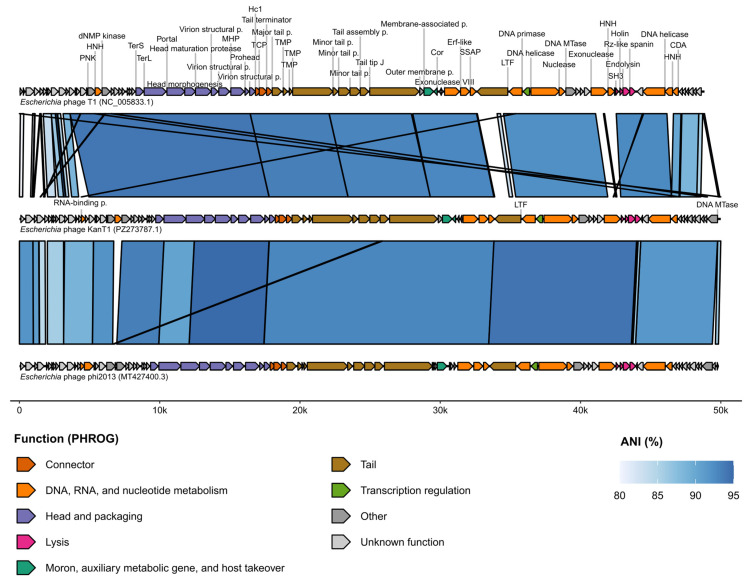
Genomic comparison of KanT1, phi2013, and T1 phage genomes. Colors represent functional annotations according to the PHROG database. Sequence identity was calculated using fastANI with a 150 bp window. Genetic maps were prepared using the gggenomes library in R. Abbreviated protein names: polynucleotide kinase, PNK; HNH endonuclease, HNH; deoxynucleoside monophosphate kinase, dNMP kinase; terminase small subunit, TerS; terminase large subunit, TerL; portal protein, Portal; major head protein, MHP; head closure Hc1, Hc1; tail completion or Neck1 protein, TCP; tail length tape measure protein, TMP; central tail fiber J, Tail tip J; Cor superinfection exclusion protein, Cor; Erf-like ssDNA annealing protein, Erf-like; single strand DNA binding protein, SSAP; tail fiber protein, LTF; DNA methyltransferase, DNA MTase; DNA repair exonuclease, Exonuclease; cytidine deaminase, CDA; DNA methyltransferase, DNA MTase. Other names may contain “p.” for “protein”.

BGI sequencing revealed that KanT1 possesses a linear double-stranded DNA genome comprising 49,498 base pairs, similar to the genomes of bacteriophages phi2013 and T1 (Figure 3). The average GC content across the genome is 45.49%. Since T1-like bacteriophages employ a headful DNA packaging mechanism and therefore lack fixed physical genome ends, the genome was oriented according to the reference bacteriophage T1 genome (GenBank ID—NC_005833.1) to enable consistent comparative genomic analysis. Gene annotation was performed using Pharokka and Phold, which identified a total of 88 open reading frames (ORFs) (Table S1) [27,28]. Whole genome alignment between KanT1, phi2013, and T1 phages (Figure 3) revealed a consistent set of encoding ORFs with occasional repositioning of HNH nucleases, consistent with the highly mobile nature of these genes [29].

It was previously reported that only ~50% of T1 genes have homologous proteins with a known function [7], and for KanT1, 43 out of 88 ORFs (~49%) have been labeled as hypothetical proteins, highlighting our lack of understanding of the bacteriophage genomic “dark matter”.

Notably, protein structure-informed annotation using Phold improved functional annotation by enabling the prediction of functions for eight proteins that remained unannotated after classical hidden Markov model (HMM)-profile-based annotation with Pharokka. These proteins were identified as a portal protein, two virion structural proteins, a prohead protein, a DNA helicase, a nuclease, and a cytidine deaminase. These proteins also remained unannotated in the T1 and phi2013 genomes after annotation with Pharokka, but were subsequently assigned by Phold and shared high sequence identity with KanT1 proteins.

Thus, structure-based annotation enables the functional prediction of homologous proteins of T1-like phages that do not exhibit high sequence similarity to reference proteins in the PHROGs database.

No tRNA genes or genes associated with known bacterial toxins—as per the Virulence Factor Database (VFDB)—were detected. Analysis with DefenseFinder did not identify any antidefense systems within the KanT1 genome. However, additional functional annotation using dbAPIS predicted a superinfection exclusion protein SieA at the locus corresponding to CDS_0027 [30]. Nevertheless, given the lack of supporting evidence from other annotation tools and the overall context of the genome, this prediction should be interpreted with caution.

To identify the proteins responsible for host recognition, we employed predictive algorithms PhageDPO and PhageRBPdetect [31,32]. This analysis converged on a high-confidence candidate for the primary host adhesion protein: the product of CDS_0062, confidently annotated as a tail fiber protein (PhageDPO score: 96%; PhageRBPdetect score: 0.99). The PhageRBPdetect tool also predicted with high confidence two other structural proteins of the phage tail apparatus: the major tail protein (CDS_0045, score: 0.98) and the central tail fiber J protein (CDS_0053, score: 0.97).

### 2.4. Comparative Genomic Analysis of the KanT1

**Figure 4:**
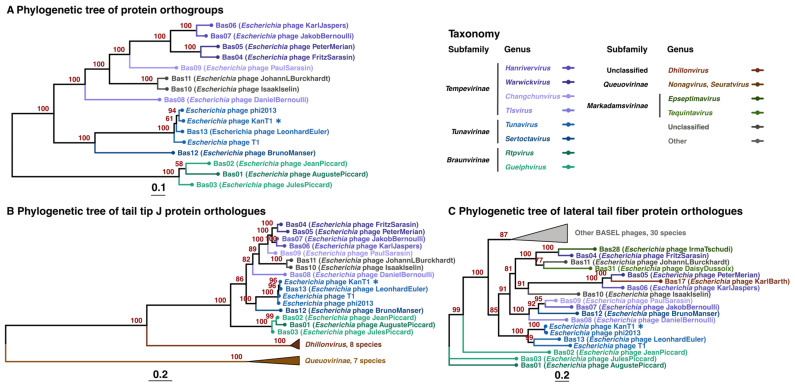
The position of KanT1 (labeled with an asterisk *) on the phylogenetic tree of bacteriophages from the *Drexlerviridae* family. (**A**) Phylogenetic tree of indicated bacteriophages based on protein orthogroups. Phylogenetic trees of central tail fiber J proteins (CDS_0053) (**B**) and lateral tail fiber proteins (CDS_0062) (**C**). Bacteriophage genera are indicated with color. Bootstrap support is provided at each node. Branch lengths reflect the number of substitutions per site.

According to a whole-genome BLASTn analysis, the closest relative of KanT1 is the Escherichia phage phi2013 (98% genome coverage, 93.25% identity; GenBank ID: MT427400.3) [33]. To further resolve its phylogenetic placement, a proteome-based tree was constructed, which positioned KanT1 within a well-supported clade of other *Tunavirus* phages, confirming its status as a novel species within this genus (Figure 4A). In addition, a whole-genome alignment of KanT1 against the BASEL *Drexlerviridae* dataset, together with phi2013 and T1, was performed using VIRIDIC (Figure S1) [34]. VIRIDIC analysis validates the assignment of KanT1 to the BASEL *Tunavirus* phages.

We further built phylogenetics trees of individual lateral tail fiber and central tail fiber J proteins (Figure 4B,C). Homologs of each protein were identified by BLASTP using the KanT1 protein as a query against the complete BASEL dataset of phages from distinct phylogenetic groups, supplemented with phi2013 and T1. This approach recovered 31 homologs of J and 54 homologs of LTF proteins. These sequences were used for the subsequent phylogenetic reconstruction of central and lateral tail fibers proteins evolution.

Across all reconstructed phylogenies, the topology of the central tail fiber protein J tree was consistent with the phage taxonomy and proteome-based tree (Figure 4B), supporting a convergent evolution of this protein with the rest of the T1-like phages proteome. While LTF proteins of the *Tunavirus* phages also form a monophyletic group, the branches corresponding to distinct phage genera did not reproduce the topology obtained for the protein J and proteome-based tree. This result suggests a higher variability of the LTF proteins, consistent with their proposed role in the recognition of divergent O-antigens and possible horizontal gene transfer events.

Collectively, these results position KanT1 as a bona fide member of the *Tunavirus* genus, retaining a conserved genomic characteristic of this group while accommodating limited gene-level variation.

### 2.5. Cor Superinfection Exclusion Locus Analysis

**Figure 5:**
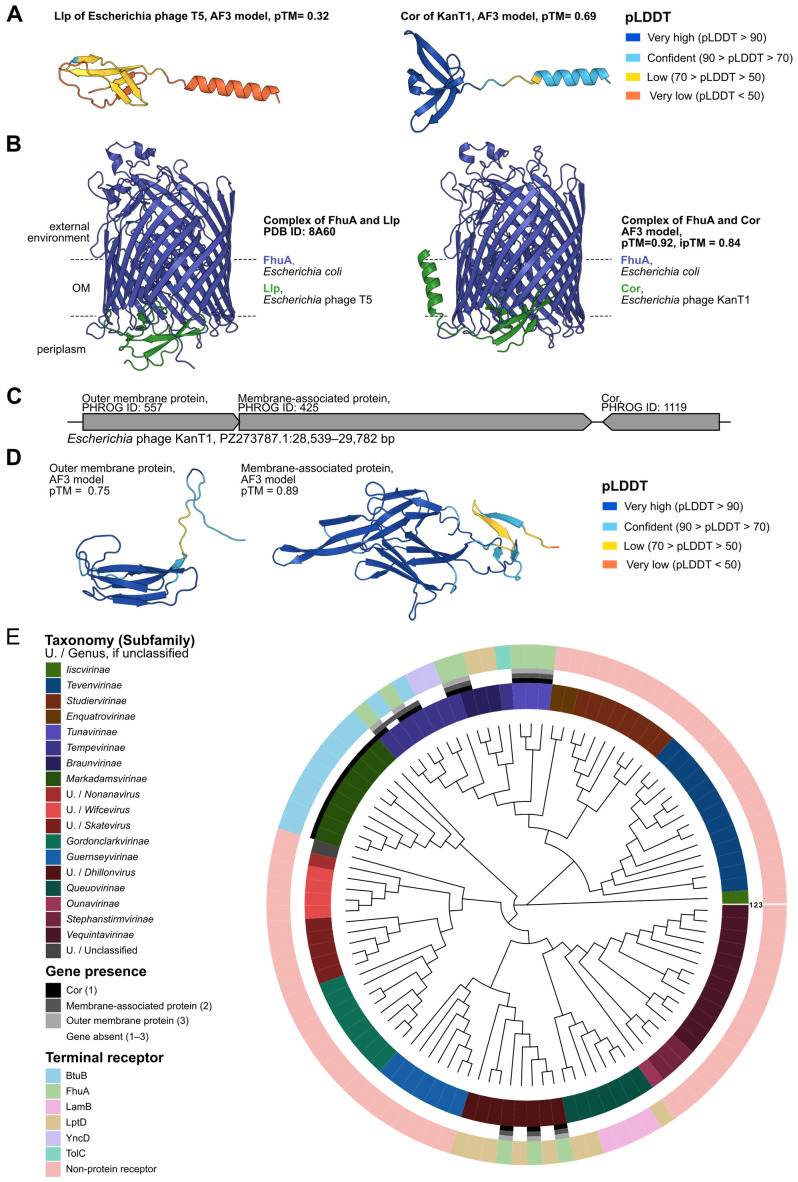
Cor cassette is a consistent feature across BASEL FhuA targeting phages. (**A**) AlphaFold3 structural prediction of Llp superinfection exclusion from and the Cor superinfection exclusion protein from KanT1. Modeled structures are colored by pLDDT, pTM values are indicated. (**B**) Crystal structure of FhuA in complex with the Llp (PDB ID: 8A60 [35]) and AF3 structural prediction of the Cor superinfection exclusion protein from KanT1 in complex FhuA (pTM and ipTM values indicated). (**C**) Locus of *Escherichia* phage KanT1 representing the Cor locus with two upstream proteins: outer membrane protein and membrane-associated protein with PHROG categories indicated. (**D**) AF3 structural prediction of outer membrane protein and membrane associated protein from KanT1. Modeled structures are colored by pLDDT, pTM values are indicated. (**E**) Phylogenetic tree of BASEL bacteriophages based on large terminase subunit (TerL) showing the distribution of the Cor protein, membrane-associated protein, and outer membrane protein. The inner ring denotes taxonomy, three central rings indicate gene presence/absence, and outer color strips denote terminal receptor types. pLDDT: per-residue confidence score from AlphaFold reflecting predicted local structural accuracy; pTM: predicted model quality (AlphaFold confidence score); ipTM: predicted accuracy of inter-chain interfaces (AlphaFold-Multimer confidence score).

In KanT1, its closest relative phi2013, and *Escherichia* phage T1, we identified a conserved genomic locus comprising a Cor superinfection exclusion protein and two upstream genes encoding a predicted membrane-associated protein and an outer membrane protein. The function of T1 Cor was reported previously and is similar to a homologous superinfection exclusion lipoprotein (Llp) from the phages T5 and BF23 [13,35,36]. Cor is an outer-membrane lipoprotein that binds FhuA, therefore blocking its accessibility for recognition by the superinfecting phages. It is speculated that Sie allows the infecting phage to safeguard host resources and precisely control the transcription program without interference from a secondary infection. In addition, the Sie mechanism prevents adsorption of the phage progeny to the remnants of the lysed cell [37]. Considering that KanT1 also exploits FhuA as a receptor, we modeled a structure of the Cor protein and its complex with FhuA (GenBank ID—BAB96726.2) using AlphaFold3 (AF3) (Figure 5B) [38].

The predicted KanT1 Cor resembles the fold of T5 Llp (Figure 5A). Initial attempts to obtain the FhuA:CorA complex docked CorA at the outer side of the protein, which is not available for CorA binding *in vivo*. However, exclusion of a flexible N-terminal FhuA region (residues 1–70) generated a highly confident prediction (ipTM = 0.84) with Cor docked at the periplasmic side of FhuA (Figure 5B), resembling a recently reported structure of the FhuA:Llp complex [35]. Additionally, the structural alignment of the Llp structure within the complex, with the CorA structure modeled in the same state, has a TM-score of 0.69, confirming similarity of their folds. The similarity of the KanT1 Cor and T5 Llp structures, as well as an overlap in their binding sites within FhuA, supports an identical Sie mechanism for different siphophage families.

Despite employing structural searches with Foldseek and HHPRED analysis, no significant hits were identified for the two proteins encoded upstream of Cor (Figure 5C,D) [39]. Considering their predicted membrane localization and association with *cor*, we assume that activity of these proteins could be related to the Sie mechanism. A review of the data presented by Wietzorrek et al. (2006) demonstrates that this locus, arranged in an identical order, is found immediately downstream of the tail fiber gene cluster in non-related phages—T1, HK022, N15, ES18, and Φ80 [12]—all relying on FhuA as a protein receptor, while the close relatives of these phages not utilizing FhuA lacked the corresponding gene cassette [12]. Another recent work reported an intriguing correlation between the *cor*-adjacent locus identity and a specificity of the host receptor for *Drexlerviridae* phages [40].

To further analyze the relationship between the *cor* cassette and the phage receptor, we utilized the genomes of phages from the BASEL collection, with experimentally validated receptors. A phylogenetic tree based on the terminase large subunit has been built and the presence of *cor* and upstream genes encoding membrane proteins have been marked together with terminal receptor identity (Figure 5E). Cor was identified in 20 phages, and in 10 instances (*Drexlerviridae* and *Dhillonvirus* phages, all utilizing FhuA receptors), the presence of membrane proteins was also confirmed. Nine other instances detected the presence of Cor-like proteins in *Markadamsvirinae* phages, without accompanying membrane proteins. There was also one species of *Tempevirinae* phages lacking the outer membrane protein, but with the present Cor and the membrane-associated protein. Notably, these phages were reported to utilize BtuB as a receptor, suggesting that the Cor/Llp Sie mechanism extends to different outer membrane proteins. Together, these results confirm previous observations of an extended *cor* cassette association with a FhuA receptor.

### 2.6. SH3 Domain Analysis

**Figure 6:**
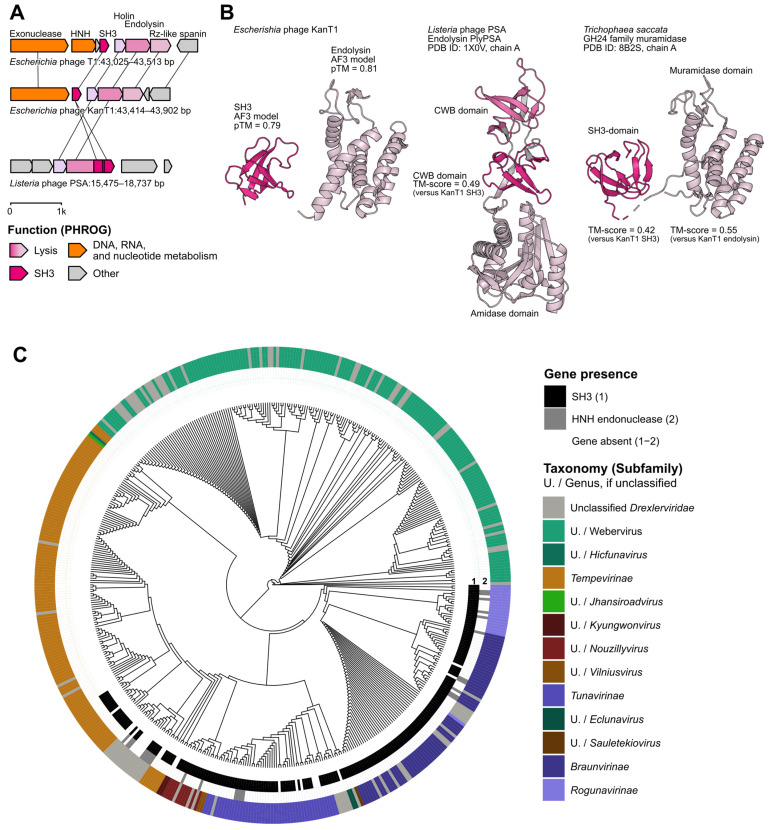
SH3 is consistently associated with the lysis cassette across *Drexlerviridae*. (**A**) Comparison of the lysis module organization in *Escherichia* phages T1, KanT1, and *Listeria* phage PSA. In *E. coli* phages, a standalone SH3 gene is located between the HNH endonuclease and endolysin genes, whereas in *Listeria* phage PSA the SH3 domain is fused to the endolysin. (**B**) Structural models of the standalone SH3 protein and endolysin from *Escherichia* phage KanT1 predicted with AF3 (pTM values indicated), and comparison with the experimentally resolved structure of the endolysin PlyPSA from *Listeria* phage PSA (PDB ID: 1X0V [44]). CWB—cell wall binding domain. Structural alignment demonstrates similarity between the standalone SH3 and the SH3 domain of PlyPSA, as well as between the catalytic muramidase/amidase domains (PDB ID: 8B2S [45]) (TM-scores indicated). ipTM: predicted accuracy of inter-chain interfaces (AlphaFold-Multimer confidence score). pTM: predicted model quality (AlphaFold confidence score). TM-score: length-independent structural similarity between two structures. (**C**) Phylogenetic tree of *Drexlerviridae* phages based on the large terminase subunit, with annotation of SH3 and HNH domain presence. The circular tree displays subfamily- or genus-level classification (colored outer ring), while inner rings indicate the presence of SH3 (black, *n* = 188) and HNH (gray, *n* = 25) domains. SH3 domains are enriched in specific clades, particularly within *Tunavirus* and related genera, suggesting lineage-specific conservation and functional importance.

In our attempts to increase KanT1 annotation quality, we manually curated all proteins predicted as “hypothetical”. Structural homology analysis with Foldseek [39] yielded a significant, high-confidence hit of a standalone SH3 domain (hereinafter SH3) encoded upstream of the cell lysis module (Figure 6A). This was corroborated by sequence-based analysis with InterPro [41], which classified the protein as a member of the DUF7244 family, of the SH3 domain clan.

In the KanT1 genome, the SH3 protein-encoding gene is located adjacent to the lysis cassette, situated between a DNA repair exonuclease and the pinholin. The lysis module demonstrates a classical architecture and is composed of pinholin, SAR endolysin, and unimolecular u-spanin. Notably, the SH3 domain, as a fusion with endolysin or as a standalone protein, is a frequent component of the lysis modules of phages infecting Gram-positive bacteria [17,18,19]. SH3 domains are widespread in nature and mediate protein recognition [42] or the recognition of peptide motifs in the bacterial cell wall. In Gram-positive bacteria, a thick peptidoglycan layer forms the main component of the cell wall and lies at the cell surface, whereas in Gram-negative bacteria a much thinner peptidoglycan layer is located in the periplasm between the inner and outer membranes. Association of SH3-like cell wall-binding domains with endolysins can enhance their activity by improving binding to peptidoglycan, and the diversity of these domains contributes to endolysin specificity and thus can influence phage host range. In addition, SH3-like domains can help to retain endolysins at the cell surface of lysed bacteria, which may modulate their diffusion and activity in the surrounding environment. SH3 domains were not reported as part of the lysis cassettes of phages infecting Gram-negative bacteria; however, considering the strong co-localization pattern, we hypothesize that T1-like SH3 proteins could modulate endolysin activity, similar to their role in phages of Gram-positive bacteria.

To analyze the variability of the lysis cassette’s genomic context, PPanGGOLiN [43] was employed on a collection of 42 *Tunavirus* genomes. The analysis revealed that the modular structure of DNA repair exonuclease–SH3–holin–endolysin–spanin cassette is highly stable and conserved across 39 of these phages. All protein families within this locus were classified by PPanGGOLiN as persistent, i.e., present in 90–99% of analyzed genomes. To extend this analysis across the entire *Drexlerviridae* family, PPanGGOLiN was used to assess the variability of the broader lysis cassette context. The core module DNA repair exonuclease–holin–endolysin–spanin was found to be a stable, persistent feature, detected in 508 out of 522 phages, with the respective protein families classified as persistent by the tool. In contrast, the SH3 protein family was classified as part of the shell genome, being present in only 188 of the 522 phages. Notably, when present, the SH3 gene was exclusively located in close proximity to the lysis module.

Next, sequence logos were generated from the multiple sequence alignment of SH3 proteins identified in the *Drexlerviridae* collection and solely in *Tunavirus* phages (Figure S2A). The resulting logos reveal a clear pattern of conservation across the domain; several positions display strong enrichment for hydrophobic and aromatic residues, which may stabilize the β-barrel structure characteristic of SH3 domains. AF3 modeling confirmed the expected minimal topology of the KanT1 SH3 domain, (Figure 6B) comparable with the classic example of the SH3 cell-wall binding domains of the Gram-positive Listeria phage PSA endolysin [44]. Interestingly, an HHPRED search of the KanT1 endolysin homologs identified a fungal muramidaze fused to a SH3 cell wall recognition domain [45], confirming the co-occurrence of SH3 and KanT1-like endolysin enzymes across kingdoms (Figure 6B).

To better understand the distribution of SH3 proteins across *Drexlerviridae* phages, we have constructed a phylogenetic tree of 522 phages from the INPHRARED collection based on the large terminase subunit (Figure 6C).

SH3 exhibited a consistent phylogenetic distribution, being found in the majority of *Tunavirinae*, *Braunvirinae*, and *Rogunavirinae* within *Drexlerviridae* (188 phages, Figure 6C). In the genome of the phage T1, the SH3 is encoded downstream of the HNH nuclease, absent in the KanT1 genome (Figure 6A). To understand if SH3 is part of a variable locus mobilized by the HNH nuclease, we mapped the HNH nuclease gene in the vicinity of the lysis cassette, which revealed a rather sporadic occurrence, not directly correlating with the SH3 gene presence. Still, a possibility remains that homing nuclease facilitated the dissemination or ancestral acquisition of SH3 gene [29].

To investigate whether phages lacking SH3 instead encode an alternative subunit associated with the lysis module, we extracted genomic loci between DNA repair exonuclease and pinholin genes and analyzed encoding protein frequency (Figure S2B).

In phages lacking the SH3 protein, the genes most frequently found immediately after the DNA repair exonuclease encoded polynucleotide kinase and deoxynucleoside monophosphate kinase (Figure S3A,B). We hypothesize that these genes represent an extension of the replication module, enhancing nucleotide supply, rather than being related to the endolysin locus. There were seven instances where these kinases were found together with the SH3 protein. The ’other’ category includes a small number of phages that are the sole representatives of their respective genera and encode unique hypothetical proteins downstream of the DNA repair exonuclease (Figure S2B). Due to the lack of close relatives and conserved annotations, these proteins could not be systematically classified.

Together, our results indicate the SH3 domain as a conserved feature of the lysis module of four subfamilies within *Drexlerviridae*. The protein is occasionally associated with HNH homing nuclease, suggesting its role in SH3 dissemination. To our knowledge, this is the first report of the SH3 domain co-localized with the lysis module of phages of Gram-negative bacteria. Considering the established role of SH3 domains in the modulation of the endolysins’ activity in phages of Gram-positive bacteria, we hypothesize that KanT1 and T1 SH3 also could be involved in cell lysis activity. Further experimental research is needed to verify this prediction and establish the biological role of this accessory module in T1-like phages.

## 3. Materials and Methods

### 3.1. Bacteria, Phages, and Growth Conditions

The following *E. coli* strains were used in this work: BW25113, HS, and ECOR collection resembling a variety in the O-antigen structures (72 strains) [23]. *E. coli* strains with the deletion of *fhuA* and *rfaG* were obtained from the KEIO collection [25]. Bacteria were routinely propagated in liquid LB media (*m*/*v*: 10% Triptone, 5% Yeast extract, 10% NaCl) at 37 °C. Bacteriophages Bas04 (*Escherichia phage FritzSarasin*), Bas07 (*Escherichia phage JakobBernoulli*), Bas08 (*Escherichia phage DanielBernoulli*), Bas12 (*Escherichia phage BrunoManser*), and Bas13 (*Escherichia phage LeonhardEuler*) were obtained from the BASEL collection [8] and were propagated as described below.

### 3.2. KanT1 Bacteriophage Isolation and Purification

KanT1 was isolated from an environmental water sample collected from the Parkoviy Stream near a zoo in Kaliningrad, Russia [54°43′20.8″ N 20°29′30.6″ E]. Water was filtered through a 0.22 μM filter (Millipore, Burlington, MA, USA), and 100 μL was mixed with the top 0.6% LB agar with *E. coli* BW25113 culture. Single plaques were re-streaked and then inoculated into 10 mL of bacterial culture pre-grown in LB to OD600 = 0.3 (~10^8^ cfu/mL). Phage infection was carried out overnight at 37 °C. Cell lysate was spun down by centrifugation (10 min at 6000× *g*) and treated with 50 µL of chloroform before storage.

For downstream genomic DNA extraction, the phage was precipitated with PEG. In short, 8 mL of lysate with titer ~10^10^ pfu/mL was treated with 2 µL DNAse I and 2 µL of RNAse A at 37 °C for 30 min to remove fragments of host DNA and RNA, and then was mixed with 2 g of PEG 8000; NaCl was adjusted in solution to 1 M. Phage particles were precipitated at +4 °C overnight with rotation and then collected by centrifugation (10 min at ~3600× *g* in bucket rotor). Precipitate was resuspended in 400 µL of STM buffer (NaCl—100 mM, MgSO_4_—10 mM, Tris-HCl (pH 7.5)—50 mM). To remove PEG, 400 µL of chloroform was added, and the mixture was rigorously vortexed for 1 min. Following centrifugation (5 min at 6000× *g*), the supernatant was collected and stored at 4 °C.

### 3.3. Transmission Electron Microscopy

For negative staining transmission electron microscopy (TEM), the formvar/carbon Cu-supported TEM grid (Ted Pella, Redding, CA, USA, catalaog number 01801) was cleaned in Ar: O_2_ plasma for 40 s (1070 Nanoclean, Fischione, Export, PA, USA). A volume of 20 μL of phage lysate was dropcasted onto the carbon side of the grid and left for 1 min. The residual sample was blotted by touching the grid with the blot paper, followed by two rinses in droplets of distilled H_2_O. After that, the grid was immediately floated on top of the drop of uranyl acetate (UA, 1 wt.% solution, 9 μL) and was held in touch with UA, droplet with tweezers for 45 s. The excess negative stain was blotted by gently sliding the side of the grid along the piece of blotting paper. The grid with the stained sample was left in the air until completely dry. Bright-field TEM images were acquired on a Titan Themis Z transmission electron microscope (Thermo Fisher Scientific, Waltham, MA, USA) operated at 200 kV using a BM-Ceta 4 K × 4 K CMOS camera (Thermo Fisher Scientific, Waltham, MA, USA) with 4 pixel binning. KanT1 virion dimensions were measured on TEM images acquired at 30,000× magnification and analyzed using ImageJ software (v1.54) [46].

### 3.4. One-Step Growth Curve

One-step growth curve assays were performed using *E. coli* BW25113 precultured in 10 mL LB to OD_600_ = 0.6 (~2 × 10^8^ CFU/mL). KanT1 phage from a 10^10^ PFU/mL stock was added to achieve an initial multiplicity of infection (MOI) of 0.001. Aliquots (1 mL) were collected every 10 min, mixed with 1:100 (*v*/*v*) chloroform, and centrifuged at 6000× *g* for 5 min to remove bacterial cells. Phage titers in the supernatants were determined by plaque assay: 10-fold dilutions were plated on pre-poured plates with 1.2% bottom LB agar and 0.6% top LB agar overlaid with a 100-fold dilution of an overnight BW25113 culture. Plates were incubated at 37 °C overnight. The experiment was performed in biological triplicate.

### 3.5. Phage Titer Determination and Efficiency of Plating (EOP) Assay

Phage titers in cell lysates were quantified using the double-layer agar overlay method. Overnight bacterial cultures (100 μL) were mixed with 10 mL of 0.6% top LB agar containing the appropriate antibiotics, then poured onto pre-poured 1.2% bottom LB agar plates. Serial 10-fold dilutions of phage lysates (10 μL spots) were applied to the top agar, allowed to absorb, and the plates were incubated at 37 °C overnight. All experiments were conducted in biological triplicate. Plaque diameters were calculated using the ImageJ software (v1.54) [46].

### 3.6. Liquid Culture Infection

To track KanT1 phage infection dynamics in liquid culture, we used an EnSpire Multimode Plate Reader (PerkinElmer, Hong Kong, China). Overnight *E. coli* cultures were diluted 100-fold into 10 mL LB and grown at 37 °C to OD600 = 0.6 (~2 × 10^8^ CFU/mL). Then, 200 μL aliquots were transferred to a 96-well plate and infected with KanT1 from a 10^10^ PFU/mL stock at the indicated MOI. Optical density was monitored continuously for 16 h. All experiments were performed in three biological replicates.

### 3.7. Determination of the Host Range

To determine the host range of KanT1, the efficiency of the plating assay was conducted using the double agar overlay method. Overnight cultures of bacteria (100 µL) were mixed with 10 mL of 0.6% top LB agar and poured on the surface of precast 1.2% bottom LB agar plates. A volume of 3 µL drops of serial 10-fold phage (10^10^ pfu/mL stock) lysate dilutions were spotted on the top agar, allowed to dry, and the plates were incubated at 37 °C overnight.

### 3.8. DNA Sequencing

Libraries for Whole Genome Sequencing were prepared from 500 ng of phage DNA using MGI Easy PCR-Free Library Prep Set (MGI Tech, Shenzhen, China), following the manufacturer’s instructions. Enzymatic fragmentation was performed according to the manufacturer’s protocol, followed by selection of 400–450 bp-long fragments on the provided DNA Clean Beads. The concentration of the prepared libraries was measured using Qubit Flex (Life Technologies, Waltham, MA, USA) with the dsDNA HS Assay Kit. The quality of the prepared libraries was assessed using 4200 TapeStation System with the High Sensitivity D1000 ScreenTape Assay (Agilent, Santa Clara, CA, USA). DNA libraries were further circularized, pooled, and sequenced using DNBSEQ-G400 in 2 × 150 bp PE mode. FastQ files were generated using the zebracall V2 software (MGI Tech).

### 3.9. Phage Genome Assembly and Annotation

At first, fastp v. 0.24.0 was implemented in order to check the quality of raw reads and trim adapters [47]. Then SPAdes v. 4.0.0 with mode --metaviral was used to assemble a genome [48]. The closest sequence similarity of the assembled phage to the phi2013 was established with the help of a whole-genome BLASTn search implemented in BLAST+ v. 2.16.0+ against the core nucleotide database [49]. Whole-genome alignment of selected sequences with KanT1 was performed with web VIRIDIC [34]. The PhageTerm v. 1.0.12 analysis did not reveal obvious genome termini and suggested the presence of terminal redundancy [50]. Phage genome annotation was primarily performed with the use of pharokka v. 1.8.0. and improved with Phold v. 1.0.0 [27,28,51]. In addition, functional annotation was complemented using dbAPIS to improve the identification of phage proteins and refine predicted gene functions [30]. Phage receptor-binding proteins (RBPs) and depolymerases were predicted with PhageRBPdetect v. 4.0.0 and PhageDPO v. 0.1.0, respectively [31,32]. DefenseFinder v. 2.0.1 was implemented to search for antidefense systems [52,53]. All mentioned tools were used with default parameters unless specified otherwise.

### 3.10. Sequence Alignment, Phylogenetics Analysis, and Structural Comparison

To determine the taxonomic position and evolutionary relationships of KanT1, a comparative genomics approach was employed. A dataset comprising all *Drexlerviridae* phages from the BASEL collection (*n* = 13, [54]), along with the additionally selected sequences, was assembled.

Phylogenetic reconstructions were performed using two complementary approaches. First, a genome-wide phylogenetic tree was inferred based on protein orthogroups, which were identified and clustered using OrthoFinder v. 2.5.5 [55].

Second, to examine the evolutionary history of host recognition components, individual phylogenetic trees were constructed for tail fiber proteins. The central tail fiber J protein was selected due to its role in targeting the terminal receptor and its relative conservation among closely related phages. For the more variable lateral tail fibers, which typically target primary receptors such as lipopolyscharide or O-antigen, homologous sequences were identified using a Protein–Protein BLAST search implemented in BLAST+ v. 2.16.0+ [49], using the KanT1 tail fiber protein as a query against the created database.

Multiple sequence alignments were generated using MUSCLE v. 5.3, and phylogenetic trees were constructed with IQ-TREE v. 3.0.1 [56,57]. Model selection was performed using the built-in ModelFinder algorithm implemented in IQ-TREE (option -m MFP), resulting in LG+F+R3; branch support was assessed using the ultrafast bootstrap approximation with 3000 replicates (-B 3000) [58,59].

### 3.11. Cor Superinfection Locus Analysis

In KanT1, phi2013, and T1, a Cor superinfection exclusion protein was identified together with two upstream proteins annotated as membrane-associated and outer-membrane-associated proteins. Structural models were generated using AlphaFold3 [38], and Foldseek [39] searches were employed to identify structural similarities among the upstream proteins against databases: BFVD [60], AFDB-PROTEOME, AFDB-SWISSPROT, AFDB50 [61], CATH50 [62], PDB100 [63]. To assess association of Cor cassette with the terminal receptor, we performed search of Cor, outer membrane protein, and membrane-associated protein across the BASEL collection of phages with known terminal receptors. HMM profiles were built based of protein sequences of KanT1, and proteins encoded in the distinct group of phages previously reported [12]. An alignment was performed with MUSCLE5 v. 5.3, HMM profiles were built with hmmbuild from HMMER v. 3.4 toolkit. The search was performed with hmmsearch with E-value threshold 0.005. Phylogenetic trees were reconstructed with IQ-TREE v. 3.0.1 [56,57]. Model selection was performed using the built-in ModelFinder algorithm implemented in IQ-TREE (option -m MFP), resulting in PMB+F+I+G4; and branch support was assessed using the ultrafast bootstrap approximation with 3000 replicates (-B 3000) [58,59].

### 3.12. SH3 Domain Analysis

The protein initially annotated as a glycosyltransferase by Phold was further examined using AlphaFold 3 structure prediction, Foldseek structural comparison, and InterPro sequence-based annotation [28,39,41].

To search the INPHARED collection (dated 1 January 2025), a HMM profile built from DUF7244 was queried using HMMER v. 3.4, and hits were retained only if they satisfied an E-value threshold of 10^−5^ and a coverage threshold of 0.5 [64,65].

To assess variation in the lysis cassette, PPanGGOLiN v. 2.2.6 was applied with the following parameters: --identity 0.4, --coverage 0.7 [43].

Multiple sequence alignment and phylogenetic tree construction were performed as described in the previous section, using the best evolutionary model, according to ModelFinder, Q.PFAM+F+I+R3.

### 3.13. Visualization of the Results

Genetic map visualization was performed in R (v. 4.3.2) using the packages tidyverse (v. 2.0.0), gggenomes (v. 1.1.2), ggplot2 (v. 3.4.4), ggnewscale (v. 0.4.9), and ggrepel (v. 0.9.5). Phylogenetic tree visualization and analysis were conducted using the R packages ggtree (v. 3.10.1), treeio (v. 1.26.0), and ape (v. 5.7), together with tidyverse (v. 2.0.0). Protein structure visualization was performed using PyMOL (v. 2.5.0). Data processing and additional plotting were carried out in Python (v. 3.10) using the libraries pandas (v. 2.1.1), NumPy (v. 1.26.0), matplotlib (v. 3.8.0), seaborn (v. 0.13.0), and plotly (v. 5.18.0). Sequence logos were plotted with gglogo (v. 0.1.5) and ggseqlogo (v. 0.1).

## 4. Conclusions

KanT1, identified as a laboratory contaminant, represents a novel member of the T1-like bacteriophages within the *Tunavirus* lineage of the *Drexlerviridae* family. The phage has a relatively broad host range and is capable of infecting at least some *E. coli* strains with O-antigens. KanT1 exhibits the conserved T1-like genomic architecture, while retaining limited accessory variability. Structure-guided annotation substantially improved genome interpretation, enabling the functional assignment of proteins that remained unresolved by sequence-based approaches alone, consistent with recent advances in structure-informed phage genomics.

A key finding of this study is the identification of an SH3 domain-containing protein co-encoded with the T1-like phages lysis cassette. Comparative analysis across *Drexlerviridae* demonstrates that this protein is widespread but not universal, and consistently localized in proximity to core lysis genes. Its genomic context and conservation pattern support its classification as a shell component of the lysis module. In light of the established role of SH3-like domains in modulating endolysin activity and cell wall interactions [17,18], we propose that this protein could function as an auxiliary factor contributing to lysis efficiency.

## Data Availability

Complete genome of the KanT1 has been deposited to the GenBank under accession PZ273787; other materials used in this work are available upon request from the lead contact, Artem Isaev.

## Supplementary Materials

The following supporting information can be downloaded at: https://www.mdpi.com/article/10.3390/ijms27093756/s1.
