# Supplementary material for: Analysis of a Novel T1-like Phage KanT1 Reveals a Standalone SH3 Domain as a Widespread Component of Drexlerviridae Cell Lysis Module
Source: Int J Mol Sci. 2026 Apr 23;27(9):3756. doi: 10.3390/ijms27093756 (PMC13164509; doi:10.3390/ijms27093756)
Supplement: Supplementary file 1 [file ijms-27-03756-s001.zip › Figure S2.pdf]

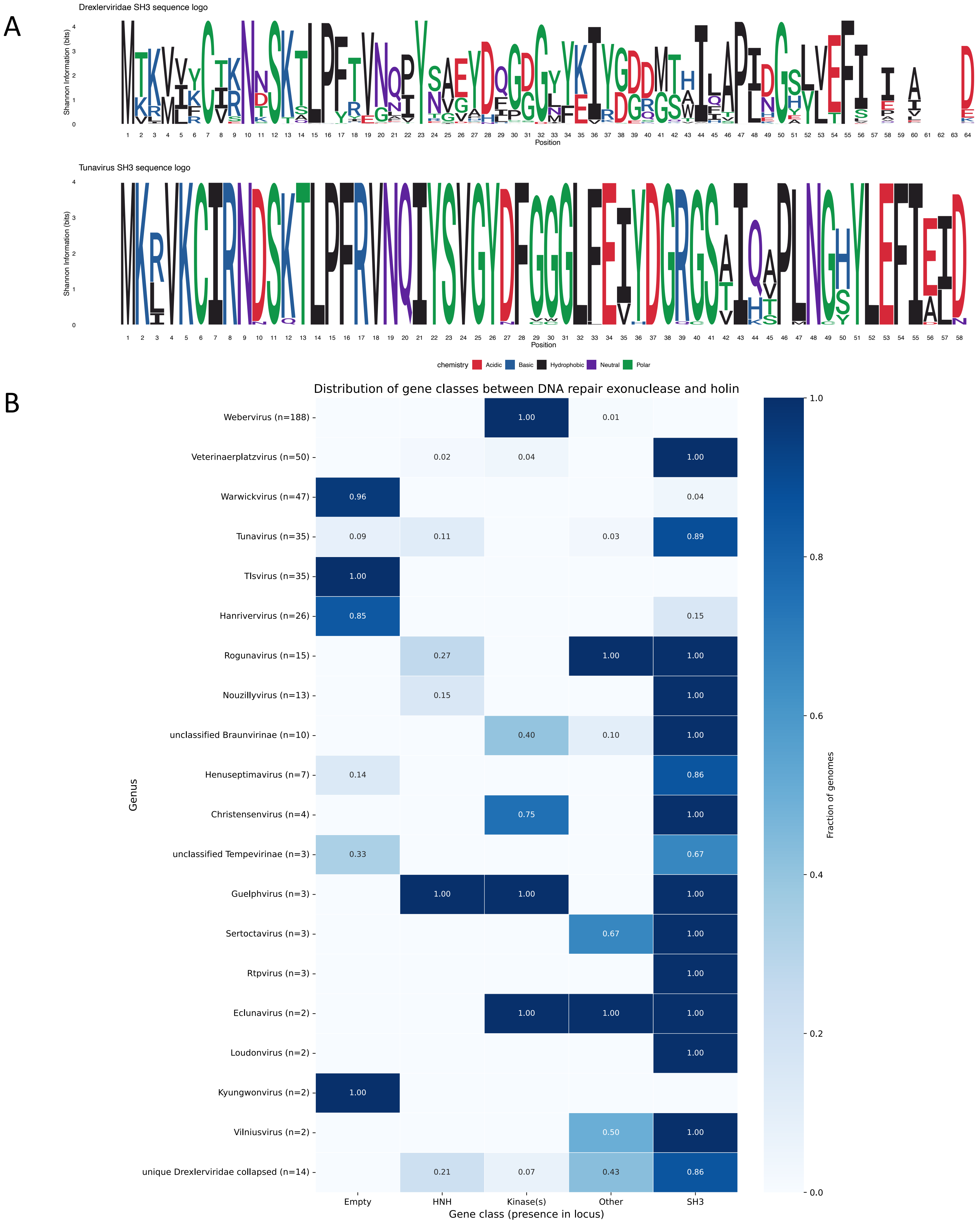

Figure S2. Sequence conservation and distribution of SH3 domain-containing proteins across Tunavirus and Drexlerviridae phages. **(A)** Sequence logo of SH3 domain-containing proteins from Drexlerviridae Tunavirus phages. The height of each letter corresponds to its frequency at a given position, measured as Shannon information (bits). Amino acids are colored according to physicochemical properties. **(B)** Heatmap showing the fraction of genomes in each genus containing specific protein families (HNH, kinases, SH3, other) or empty locus between DNA repair exonuclease and holin.
