# Supplementary material for: Analysis of a Novel T1-like Phage KanT1 Reveals a Standalone SH3 Domain as a Widespread Component of Drexlerviridae Cell Lysis Module
Source: Int J Mol Sci. 2026 Apr 23;27(9):3756. doi: 10.3390/ijms27093756 (PMC13164509; doi:10.3390/ijms27093756)
Supplement: Supplementary file 1 [file ijms-27-03756-s001.zip › Figure S3.pdf]

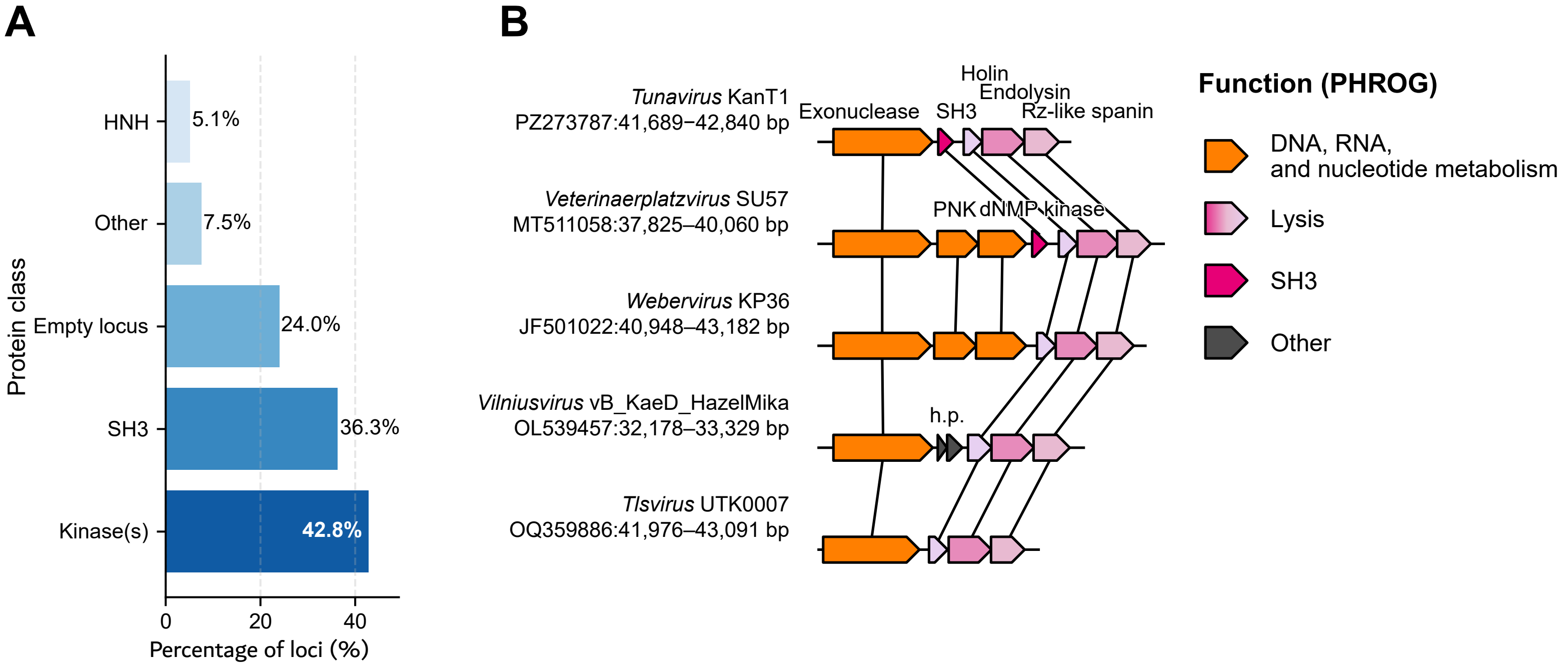

Figure S3. Analysis of gene of SH3 colocalization with lysis cassette region across *Drexlerviridae* phages. (A) Distribution of the loci content between DNA repair exonuclease and lysis cassette in analyzed phages. ‘Empty’ stands for holin after the exonuclease. (B) Representative genomic regions from selected genera illustrating variability of the analyzed locus. Genes colored according to their predicted function. h.p. stands for hypothetical protein.
