# Supplementary figures and images for: Analysis of a Novel T1-like Phage KanT1 Reveals a Standalone SH3 Domain as a Widespread Component of Drexlerviridae Cell Lysis Module
Source: Int J Mol Sci. 2026 Apr 23;27(9):3756. doi: 10.3390/ijms27093756 (PMC13164509; doi:10.3390/ijms27093756)

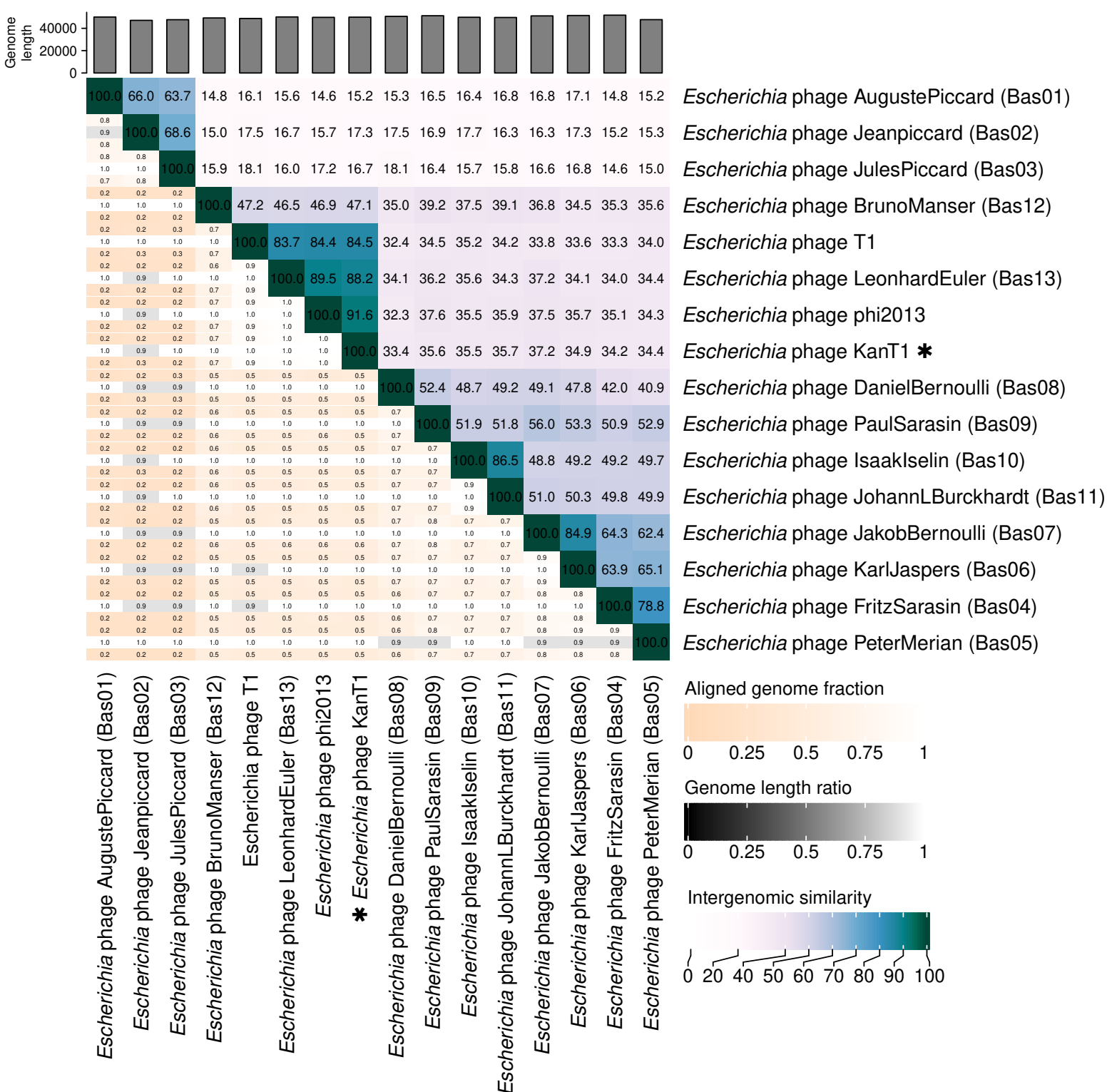

**Figure S1.** VIRIDIC analysis of BASEL *Drexelviriidae* with KanT1, phi2013, and T1.

Supplement: Supplementary file 1 [file ijms-27-03756-s001.zip › Figure S1.pdf]
